# LncRNA LINC00680 Acts as a Competing Endogenous RNA and Is Associated With the Severity of Myasthennia Gravis

**DOI:** 10.3389/fneur.2022.833062

**Published:** 2022-06-21

**Authors:** Li Liu, Huixue Zhang, Xiaoyu Lu, Lifang Li, Tianfeng Wang, Shuang Li, Xu Wang, Si Xu, Lei Li, Qian Li, Tingting Yi, Tao Wu, Zhimin Chen, Hongyu Gao, Jianjian Wang, Lihua Wang

**Affiliations:** ^1^Department of Neurology, The Second Affiliated Hospital of Harbin Medical University, Harbin, China; ^2^Department of Neurology, Heilongjiang Provincial Hospital, Harbin, China; ^3^Department of Neurology, Xuanwu Hospital of Capital Medical University, Beijing, China

**Keywords:** LINC00680, severity, myasthenia gravis, competing endogenous RNA (ceRNA), biomarker

## Abstract

**Background and Purpose:**

Myasthenia gravis (MG) is a T cell-dependent antibody-mediated autoimmune disorder that can seriously affect patients' quality of life. However, few studies have focused on the severity of MG. Moreover, existing therapeutic efforts, including those targeting biomarkers for MG, remain unsatisfactory. Therefore, it is vital that we investigate the pathogenesis of MG and identify new biomarkers that can not only evaluate the severity of the disease but also serve as potential therapeutic targets. Long noncoding RNA LINC00680 has been found to be associated with the progression of a variety of diseases as a competing endogenous RNA (ceRNA). However, the specific role of LINC00680 in MG has yet to be clarified. Here, we aimed to investigate the association between LINC00680 and the severity of MG.

**Methods:**

Bioinformatics tools, quantitative real-time PCR, Western blotting, and luciferase assays were selected to investigate key signaling pathways and RNA expression in patients with MG. The Quantitative MG Score scale and the MG Composite scale were used to evaluate the severity of MG in the included patients. Cell viability assays and flow cytometry analysis were selected to analyze cell proliferation and apoptosis.

**Results:**

Compared with control subjects, the expression levels of LINC00680 and mitogen-activated protein kinase 1 (MAPK1) in peripheral blood mononuclear cells of patients with MG were both upregulated; the levels of miR-320a were downregulated. A positive correlation was detected between LINC00680 expression and the severity of MG. Luciferase reporter assays identified that LINC00680 acts as a target for miR-320a. The *in vitro* analysis confirmed that LINC00680 regulates the expression of MAPK1 by sponging miR-320a. Finally, the functional analysis indicated that LINC00680 promoted Jurkat cell proliferation and inhibited cellular apoptosis by sponging miR-320a.

**Conclusion:**

LINC00680 may be associated with the severity of MG as a ceRNA by sponging miR-320a to upregulate MAPK1. These findings suggest that LINC00680 may represent a potential biomarker which evaluates the severity of MG and may serve as a therapeutic target.

## Introduction

Myasthenia gravis (MG) is a T cell-dependent antibody- and complement-mediated autoimmune disease that affects the function of neuromuscular junctions (1). The manifestation of this disease is a fluctuating weakness of the skeletal muscles (2–4). This weakness occurs proximally more often than distally and can be localized or systemic. Furthermore, the eye muscles are almost always affected by muscle weakness and are accompanied by diplopia and ptosis (3). The most typical characteristic of MG is that the weakness becomes more apparent with exercise and a repeated use of the muscles (fatigue) and varies at different times of day; in fact, strength is normal in the morning and weaker in the afternoon or evening (5). Various types of antibodies are involved, predominantly acetylcholine receptor (AChR) antibodies, muscle-specific kinase antibodies, and lipoprotein receptor-related protein 4 antibodies; of these, acetylcholine receptor antibodies are the most important (6). Anti-AChR antibody has a high-affinity and pathogenic immunoglobulin G (IgG); the synthesis of this antibody requires interaction between activated CD4+ T cells and B cells. CD4+ T cells, and their related cytokines, are critical for the progression of MG symptoms (7). Cytokines are crucial to the production of autoantibodies and cellular immune regulation of MG. Proinflammatory cytokines secreted by T-helper 1 (Th1) cells, such as interleukin-2 (IL-2), interferon-γ (IFN-γ), and tumor necrosis factor-α (TNF-α), are responsible for the differentiation and growth of B cells that synthesize immunoglobulin isotypes (7–10). In addition, IFN-γ can also stimulate major histocompatibility complex (MHC) class II molecule expression on the membrane of muscle cells, which facilitates the presentation of muscle cells AChR (7). Some of the cytokines secreted by Th2 cells, such as IL-4 and IL-10, are vital factors that can also affect the differentiation and growth of B cells and stimulate an immune response (11). According to statistical surveys, the annual incidence of MG is 8–10 per 1 million people and the prevalence is 150–250 per 1 million people (12). MG can have serious effects on the quality of patients' life. Nevertheless, so far, there is a notable lack of biomarkers to evaluate the clinical severity of MG; moreover, the efficacy of clinical treatments is not satisfactory (13, 14). MG is still associated with a high recurrence rate; the condition of many patients continues to worsen over time (15). Therefore, it is vital for us to investigate the specific mechanisms involved and identify new biomarkers that not only can evaluate the severity of MG but can also be used as therapeutic targets. Recent studies have reported the fact that many non-coding RNAs (ncRNAs) have been involved in the regulation of genes during the disease process in the immune system and their role was quite vital (16). This offers a new direction for identifying new markers for MG.

Long non-coding RNAs (lncRNAs), as a widely studied form of ncRNA, are a type of RNA molecule with a length of no <200 nucleotides and have no or little protein-coding function (17). Studies have shown that lncRNAs exert regulatory functions in a diverse array of biological processes, and in mechanisms of disease; these effects are known to be associated with the subcellular localization of lncRNAs. In general, cytoplasmic lncRNAs mainly regulate posttranscriptional events, while nuclear lncRNA modulates transcriptional processes (18, 19). Studies suggest that lncRNA functions *via* three mechanisms: interaction with other RNAs, interaction with chromatin, and interaction with proteins (20). Furthermore, some studies have also shown the fact that lncRNAs have been involved in the occurrence and development of immune system disorders such as multiple sclerosis (21), ankylosing spondylitis (22), and systemic lupus erythematosus (23). Besides, it has been reported that lncRNA IFNG-AS affects the activity of CD4+ T cells by influencing HLA-DRB1 expression in MG (24). Another study has shown that lncRNA XLOC_003810 promotes T-cell activation and inhibits programmed death-1/programmed death ligand-1 (PD-1/PD-L1) expression in patients with MG-related thymoma (25).

Over the past few years, more and more studies have investigated the dysregulation of the lncRNA-microRNA (miRNA)-messenger RNA (mRNA) network according to the competing endogenous RNA (ceRNA) theory. The ceRNA studies have revealed a new mechanism for RNA–RNA interactions, communication, and coregulation, in which miRNAs can affect gene expression by binding to mRNAs. The study has shown that miRNAs bind to partial complementarity sequences within transcripts of target RNA by miRNA recognition elements (MREs). Furthermore, lncRNAs could regulate the activity of miRNAs on their target mRNAs by acting as sponges for miRNAs *via* the MREs (26). As a ceRNA, lncRNA competes with mRNA target to bind to miRNA so as to reduce the inhibiting effect of miRNA on mRNA target in a great diversity of diseases (27, 28). For example, the lncRNA JPX transcript, XIST activator (JPX) was shown to upregulate the Twist1 expression by sponging miRNA-33a-5p to regulate the growth and metastasis of lung cancer (29). The lncRNA BCRT1 acts as ceRNA and regulates polypyrimidine tract binding protein 3 (PTBP3) expression by targeting miR-1303 in breast carcinoma (30). However, the specific effects of lncRNAs as ceRNAs in MG are still largely unknown. LINC00680 is a newly discovered lncRNA that is mainly localized to the cytoplasm of cells, as determined by RNALocate (http://www.rna-society.org/rnalocate/) (31). Indeed, an increasing body of evidence now indicates that LINC00680 serves as a ceRNA and is related to the development of a variety of carcinomas. For example, LINC00680 activates AKT serine/threonine kinase 3 (AKT3) by sponging miR-568, thus promoting stemness properties and decreasing chemosensitivity in hepatocellular carcinoma stemness (HCCs) (32). LINC00680 functions as a sponge of miR-410-3p to enhance high mobility group box 1 (HMGB1) expression to promote the progression of non-small cell lung cancer (33). The specific role of LINC00680 in MG is still not clarified.

In this study, we systematically identified the changes of LINC00680 expression in patients with MG and compared the expression with those from control subjects; this allowed us to correlate expression changes with the severity of MG. Then, we predicted the LINC00680-miR-320a-MAPK1 ceRNA network through bioinformatics tools and by reviewing reliable studies. Finally, biological experiments were designed to confirm the existence of the LINC00680-miR-320a-MAPK1 ceRNA network. Our results highlight the novel role of LINC00680 as a ceRNA and show that this form of LINC00680 is associated with the severity of MG by sponging miR-320a and upregulating MAPK1, thus providing new insights into the specific roles of LINC00680 in MG.

## Materials and Methods

### Patients and Clinical Data

In this study, blood samples were taken from a cohort of patients with MG (*n* = 31; 18 women and 13 men) and normal control volunteers (*n* = 31; 16 women and 15 men) who attended the Second Affiliated Hospital of Harbin Medical University. All the patients met the criteria for diagnosing MG. We excluded patients with a history of infectious disease and other associated active autoimmune diseases, patients receiving treatment with corticosteroids, immunosuppressants, intravenous immunoglobulins, or plasma exchange 1 month before consultation, pregnant women, and patients with psychiatric comorbidities or cognitive conditions that prevented the measurement of the key parameters being evaluated. The mean age of the MG group was 55.71 ± 16.13 years, while the mean age of the control group was 56.29 ± 12.88 years. An age of 50 years was used as a threshold to distinguish early-onset MG (EOMG) (≤ 50 years) from late-onset MG (LOMG) (>50 years). A part of the clinical data of all the patients and healthy controls are shown in Table 1. The Ethics Committee of the Second Affiliated Hospital of Harbin Medical University approved this study. All the subjects provided written informed consent. This study followed the provisions of the Declaration of the World Medical Association in Helsinki.

**Table 1:** Characteristics of patients with myasthenia gravis (MG) and healthy controls.

**Characteristic**	**MG** **(*n* = 31)**	**control** **(*n* = 31)**
Age (y)	55.71 ± 16.13	56.29 ±12.88
Gender (M/F)	13/18	15/16
Age of onset (y)		
EOMG(≤ 50 y)	12	–
LOMG(>50 y)	19	–
AChR Ab (Positive/Total)	20/24	–
Thymoma		
Yes	13	–
No	18	–
Subgroups		
OMG	14	–
GMG	17	–
History of the disease	No	No
Infectious disease	No	No
Other associated active autoimmune diseases		
Treatment in 1 month prior to consultation		
Corticosteroids	NO	NO
Immunosuppressants	No	NO
Intravenous immunoglobulins	No	NO
Plasma exchange	No	NO

*Among the 31 patients with MG, 24 patients had MG-related antibody test and 7 patients refused MG-related antibody test*.

Data relating to the age at onset, age, gender, and AChR Ab were collected from all the subjects. Detailed neurological examinations were performed using the Quantitative MG Score (QMGs) scale and the MG Composite (MGC) scale. The detailed information on the severity of patients with MG is shown in Supplementary Table 1.

### Clinical Samples

Peripheral blood was collected from all the subjects in a test tube containing ethylenediaminetetraacetic acid (EDTA), and a lymphocyte separation solution was used to distill mononuclear cells from the peripheral blood mononuclear cells (PBMCs). Then, the cells were kept in a refrigerator at −80°C for subsequent use.

### Bioinformatics Analysis

StarBase v2.0 (http://starbase.sysu.edu.cn/) (34) and DIANA-LncBase (http://carolina.imis.athena-innovation.gr/diana_tools/web/index.ph) (35) were used to predict the miRNAs that might bind to the identified lncRNA. The Nervous System Disease NcRNAome Atlas (NSDNA) prioritization tool (http://bio-bigdata.hrbmu.edu.cn/nsdna/search.jsp) (36) was also used to identify specific miRNAs that may be associated with MG.

### RNA Extraction, Reverse Transcription, and qRT-PCR

Trizol Reagent (Sigma Life Science, Darmstadt, Germany) was selected to distill total RNAs from PBMCs referring to the manual. The Transcriptor First Strand cDNA Synthesis Kit (Roche, Basel, Switzerland) was selected to reverse-transcribed total RNA with corresponding primers referring to the manual; this allowed us to detect the expression levels of LINC00680 and MAPK1. The miRcute Plus miRNA First-Strand cDNA Kit (Tiangen Biotech, Beijing, China) was selected to reverse-transcribed total RNA using corresponding primers and referring to the manual for the subsequent detection of miR-320a expression levels. The FastStar Universal SYBR Green Master Kit (Roche, Basel, Switzerland) was then used to detect the expression levels of LINC00680 and MAPK1 by quantitative real-time PCR (qRT-PCR). The miR-320a expression level was measured by qRT-PCR using the miRcute Plus miRNA qPCR Kit and SYBR Green (Tiangen Biotech, Beijing, China). We choose glyceraldehyde-3-phosphate dehydrogenase (GAPDH), U6 was used as an internal control, and target gene expression levels were normalized by the 2^−ΔΔ*CT*^ method. The sequences of primers are given in Table 2.

**Table 2:** The primers used for real-time PCR.

**Name Sequences**
LINC00680 Forward primer (5′->3′) GTGGAACCTCAGGCATCCA
LINC00680 Reverse primer (5′->3′) TATACACAGAGAGGGAGAAAGAC
miR-320a Forward primer (5′->3′) AAAAGCUGGGUUGAGAGGGCGA
GAPDH Forward primer (5′->3′) GAGAAGTATGACAACAGCCTCAA
GAPDH Reverse primer (5′->3′) GCCATCACGCCACAGTTT

### Cell Culture

Jurkat cells line and 293T cells line were both purchased from the American Type Culture Collection (Manassas, VA, USA), grown in a basic Roswell Park Memorial Institute (RPMI) 1640 medium (Thermo Fisher Scientific, Beijing, China) containing 10% serum of fetal bovine serum (Excell Bio, Suzhou, China) and 1% penicillin/streptomycin (Beyotime Biotechnology, Nanjing, China), and cultured in a suitable incubator (37°C, 5% CO_2_, and saturated humidity). The fresh medium was used to replace the old medium every 1 or 2 days according to the status of cell growth; cells that were in the logarithmic growth stage were used to conduct the subsequent experiments.

### Cell Transfection

A negative control (NC), miR-320a mimics, and miR-320a inhibitor were obtained from General Biol (Anhui, China) and transfected into Jurkat cells using Lipofectamine^®^ 2000 (Invitrogen, Carlsbad, CA, USA) referring to the manual. Then, a lncRNA Smart Silencer for human LINC00680 was purchased from Ribobio (Guangdong, China). Lipofectamine^®^ 2000 was selected to transfect Jurkat cells with specific plasmids, as described previously. The sense and antisense sequences for the LINC00680 Smart Silencers were as follows: siLINC00680-1, 5′-TCCATTCATTGGGAAATCA-3′ and 5′-AGGGCAGTGTGGAGTGACA-3′; siLINC00680-2, 5′-CATGGACAATATCATAGTT-3′ and 5′-CCTCAGCTCTCCATGGCTCT-3′; and siLINC00680-3, 5′-AGTGTGGAGTGACAGGCACG-3′ and 5′-AAGCATCCATTCATTGGGAA-3′. Cells were cultured in a humidified atmosphere of 5% CO_2_ at 37°C. Total RNA was then extracted from the transfected cells and analyzed by qRT-PCR to assess the transfection efficiency.

### Dual-Luciferase Reporter Assays and Cell Culture

Established protocols were used to perform a dual-luciferase reporter assay to investigate the binding activity between LINC00680 and miR-320a in 293T cells. The binding site for LINC00680 and miR-320a was predicted by the bioinformatics website (http://starbase.sysu.edu.cn/). To construct a LINC00680-WT luciferase reporter vector, we cloned a fragment of LINC00680 that contains the predicted binding site of miR-320a into the PHY-811 vector (Hanyi Biotechnology, Shanghai, China). Next, we mutated the assumed binding site for miR-320a in LINC00680, creating a vector that we named LINC00680-MUT. The LINC00680-WT or LINC00680-MUT vector was then transfected into 293T cells in combination with negative control or miR-320a mimics using Lipofectamine^®^ 2000. After 48 h of transfection, luciferase activity was determined using a dual-luciferase reporting analysis system (Promega, WI, USA) referring to the manual. Relative luciferase activity was measured and normalized to renin luciferase activity. These experiments were replicated three times in total.

### Western Blot Analysis

Protease inhibitors (Beyotime Biotechnology, Nanjing, China) and radio immunoprecipitation assay (RIPA) buffer (Beyotime Biotechnology, Nanjing, China) were used to distill total protein from Jurkat cells. Total protein concentration was measured using the Bicinchoninic Acid Protein Assay Kit (Beyotime Biotechnology, Nanjing, China). Protein extracts were then dissolved by 10% sodium dodecyl sulfate-polyacrylamide gel electrophoresis and then transferred onto polyvinylidene fluoride (PVDF) membrane (Millipore, MA, USA) by a semidry transfer way. Next, the membranes were blocked by a sealed liquid at room temperature for 0.5 h and then washed with Tris-buffered saline-Tween-20 (TBST). Then, the membranes were incubated with MAPK1 (1:1,000) and GAPDH (1:1,000) primary antibodies at 4°C overnight. The following morning, the membranes were washed with TBST and incubated with horseradish peroxidase-labeled secondary antibody immunoglobulin G (IgG) (1:1,000) for 2 h at room temperature. The PVDF membranes were then rewashed in TBST, and the Enhanced Chemiluminescence (ECL) Kit (Beyotime Biotechnology, Nanjing, China) was used to visualize positive immunobinding. GAPDH was selected as the internal control. These experiments were replicated three times in total.

### CCK-8 Assays

Cell Counting Kit-8 (CCK8) assays (Dojindo, Tokyo, Japan) were selected to measure cell proliferation. Cells that have been transfected with siLINC00680, negative control, and siLINC00680, along with miR-320a inhibitor were grown at a density of 1,500 cells/well in 96-well plates and incubated in an atmosphere containing 5% CO_2_ at 37°C and with saturated humidity. Then, 10 μl of CCK-8 reagent was added to each well at 24, 48, 72, and 96 h after transfection and incubated at 37°C for 2 h. The absorbance at 450 nm was then detected from each well. These experiments were repeated independently in triplicate.

### Flow Cytometry Analysis

Cell apoptosis was determined with the Annexin V-fluorescein isothiocyanate (FITC)/propidium iodide (PI) Apoptosis Detection Kit (BD Biosciences, San Jose, CA, USA) in accordance with the instructions. Jurkat cells were transfected with negative control, siLINC00680, and siLINC00680, along with a miR-320a inhibitor, and were cultured in a six-well plate at 5% CO_2_ at 37°C and with saturated humidity for 48 h. Subsequently, the cells were collected and washed with phosphate-buffered saline. Then, the cells were stained with annexin V-FITC and PI in the dark and the stained cells were measured by flow cytometry and analyzed by CellQuest software. These experiments were repeated in triplicate.

### Statistical Analysis

Experiments were replicated three times in total. SPSS software version 23.0 and GraphPad Prism version 8.0 were selected for statistical analyses. The continuous variables were shown as mean ± SD. Comparisons between the two groups were analyzed by the Student's *t*-test, and the multiple groups were analyzed by ANOVA. We also performed Pearson's correlation analysis. The *p*-value <0.05 indicated statistical significance.

## Results

### LINC00680 Was Upregulated in MG and Was Associated With the Severity of MG

**Figure 1:**
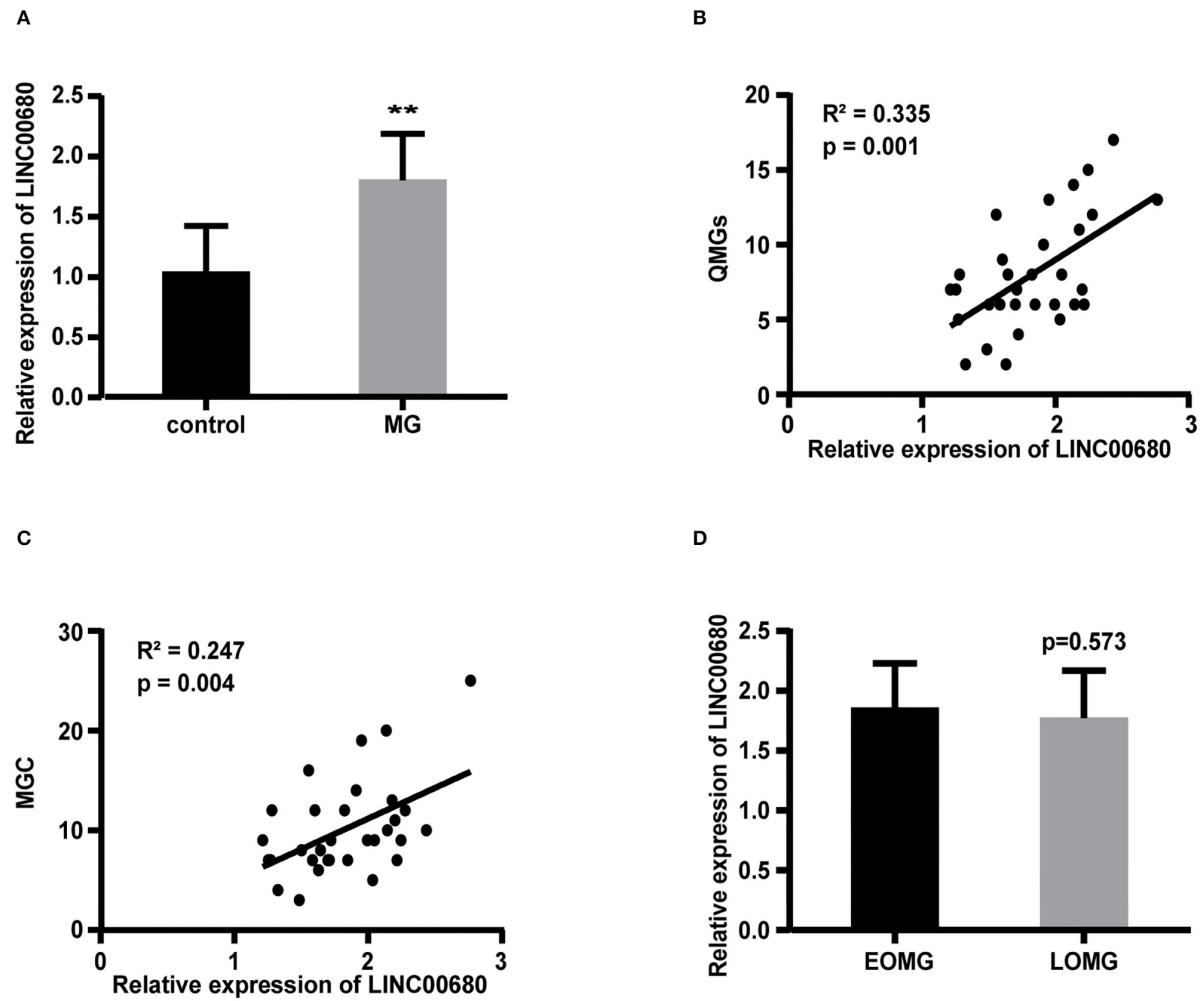
Expression of LINC00680 in MG and its correlation with MG severity and onset age. **(A)** LINC00680 expression was investigated in 31 patients with MG and 31 control subjects by real-time PCR **(B)** Correlation analyses between QMGs and LINC00680 expression in patients with MG **(C)** Correlation analyses between MGC and LINC00680 expression in patients with MG **(D)** LINC00680 expression did not differ significantly between early-onset MG (EOMG) and late-onset MG (LOMG) (^**^p < 0.01). MG, myasthenia gravis; QMGs, Quantitative MG score; MGC, Myasthenia Gravis Composite.

Quantitative real-time PCR (qRT-PCR) was selected to measure the LINC00680 expression levels in PBMCs of patients with MG and normal controls. Our results demonstrated that LINC000680 expression of patients with MG was at notably higher levels than that of normal controls (*p* < 0.01; Figure 1A). In addition, we investigated the association between the expression of LINC00680 and the severity of MG. There was a correlation between higher expression levels of LINC00680 with higher scores on the QMGs (*R*^2^ = 0.335, *p* = 0.001; Figure 1B). Moreover, the LINC00680 expression levels were also correlated with the MGC score (*R*^2^ = 0.247, *p* = 0.004; Figure 1C). However, no obvious difference was found between EOMG and LOMG with regard to the expression levels of LINC00680 (*p* = 0.573; Figure 1D).

### Construction of a LINC00680-miR-320a-Mitogen-Activated Protein Kinase 1 Interaction Network in MG

**Figure 2:**
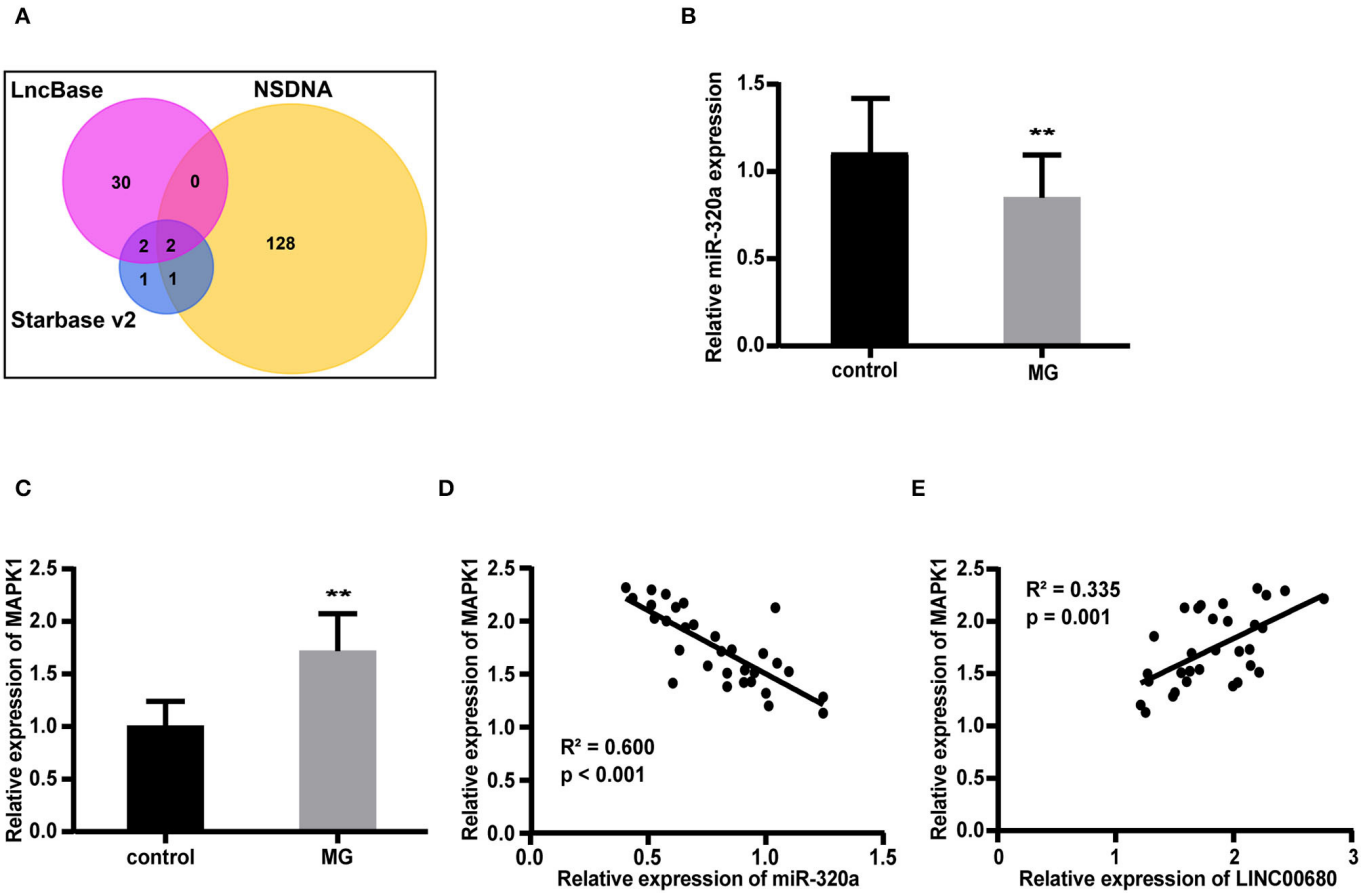
Construction of a LINC00680-miR-320a-mitogen-activated protein kinase 1 (MAPK1) interaction network for MG. **(A)** Venn diagram showing the overlapping target genes of LINC00680 associated with MG that was predicted using the Starbase v2.0, DIANA-LncBase, and the Nervous System Disease NcRNAome Atlas (NSDNA) databases **(B,C)** miR-320a expression and MAPK1 messenger RNA (mRNA) expression were examined in 31 patients with MG and 31 control subjects by real-time PCR **(D)** Correlation analyses between MAPK1 messenger RNA (mRNA) expression and miR-320a expression in patients with MG **(E)** Correlation analyses between MAPK1 mRNA expression and LINC00680 expression in patients with MG (**p < 0.01).

We predicted 34 miRNAs associated with LINC00680 through the DIANA-LncBase and 6 miRNAs associated with LINC00680 through the Starbase. We also identified 131 miRNAs that were associated with MG from the NSDNA. We identified two miRNAs at the intersection of these three sets of miRNAs; miR-320a was one of these two miRNAs (Figure 2A). A previous study reported that miR-320a was not only downregulated in patients with MG but was also mediated by the regulation of MAPK1 by directly targeting MAPK1 in Jurkat cells (1). In addition, the targeting relationship between miR-320 and MAPK1 has been verified by dual-luciferase reporter assays in a previous study (1). Therefore, we selected miR-320a and MAPK1 to construct an interaction network with LINC00680. In this study, the miR-320a and MAPK1 expression levels were also analyzed by qRT-PCR in patients with MG and normal controls. We discovered that miR-320a was downregulated and MAPK1 was upregulated in MG (*p* < 0.01, Figure 2B; *p* < 0.01, Figure 2C); these results were consistent with the previous study (1). In addition, we detected the association between the expression levels of miR-320a and MAPK1 and the association between the expression levels of LINC00680 and MAPK1 in patients with MG. We found that there was a negative correlation between miR-320a and MAPK1 and a positive correlation between LINC00680 and MAPK1, further confirming the interaction network we had identified (*R*^2^ = 0.600, *p* < 0.001, Figure 2D *R*^2^ = 0.335, *p* = 0.001, Figure 2E).

### LINC00680 Represents a Target for miR-320a

**Figure 3:**
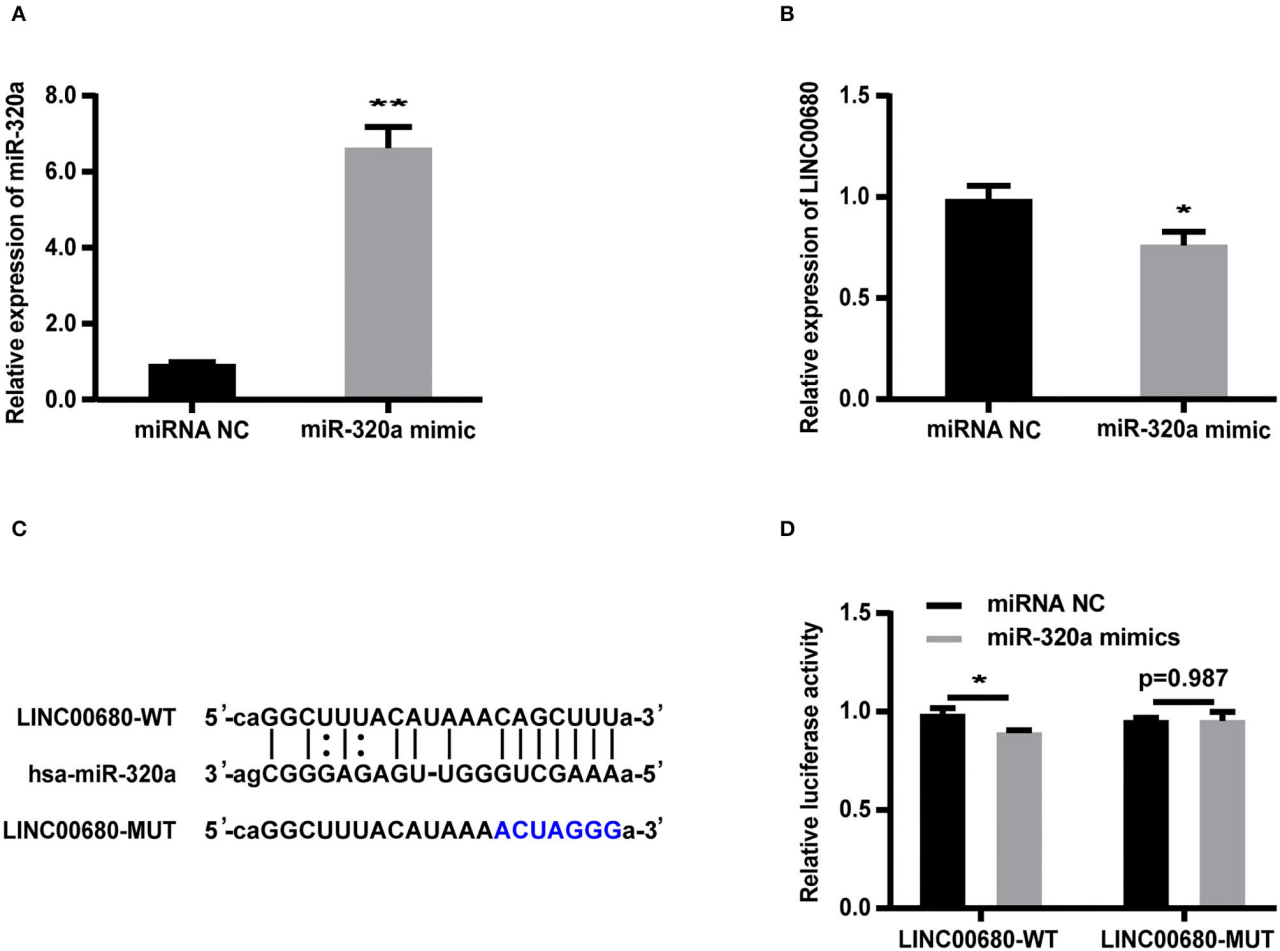
LINC00680 is a target of miR-320a. **(A)** The transfection efficiency of the miR-320a mimic was detected by real-time PCR **(B)** The relative expression levels of LINC00680 transfected with miRNA NC or the miR-320a mimic in Jurkat cells for 48 h were detected by real-time PCR **(C)** The putative miR-320a binding sequence of the wild-type and mutated sequence of LINC00680 **(D)** A luciferase reporter plasmid containing LINC00680-WT or LINC00680-MUT was co-transfected with the miR-320a mimic or miRNA NC into HEK293T cells for 48 h. Luciferase activities were calculated as the ratio of Firefly/Renilla activities (*p < 0.05, **p < 0.01).

Bioinformatics analysis revealed that the LINC00680 sequence contained a hypothetical miR-320a-binding region. To investigate the relationship between miR-320a and LINC00680, we transfected Jurkat cells with a miR-320a mimic. qRT-PCR was selected to detect transfection efficiency (*p* < 0.01; Figure 3A). The miR-320a overexpression inhibited the expression of LINC00680 (*p* < 0.05; Figure 3B). To identify the precise interaction between LINC00680 and miR-320a, we constructed LINC00680 wild-type (WT) and LINC00680 mutant (MUT) luciferase reporter vectors (Figure 3C). LINC00680-wt or LINC00680-mut and miR-320a mimic or negative control were co-transfected into 293T cells. Dual-luciferase reporter assays demonstrated that the luciferase activity of LINC00680-WT was repressed by the miR-320a mimic, although the luciferase activity of the LINC00680-MUT was not affected (Figure 3D). These findings indicated that LINC00680 is the target of miR-320a.

### LINC00680 Modulated MAPK1 Expression by Sponging miR-320a

**Figure 4:**
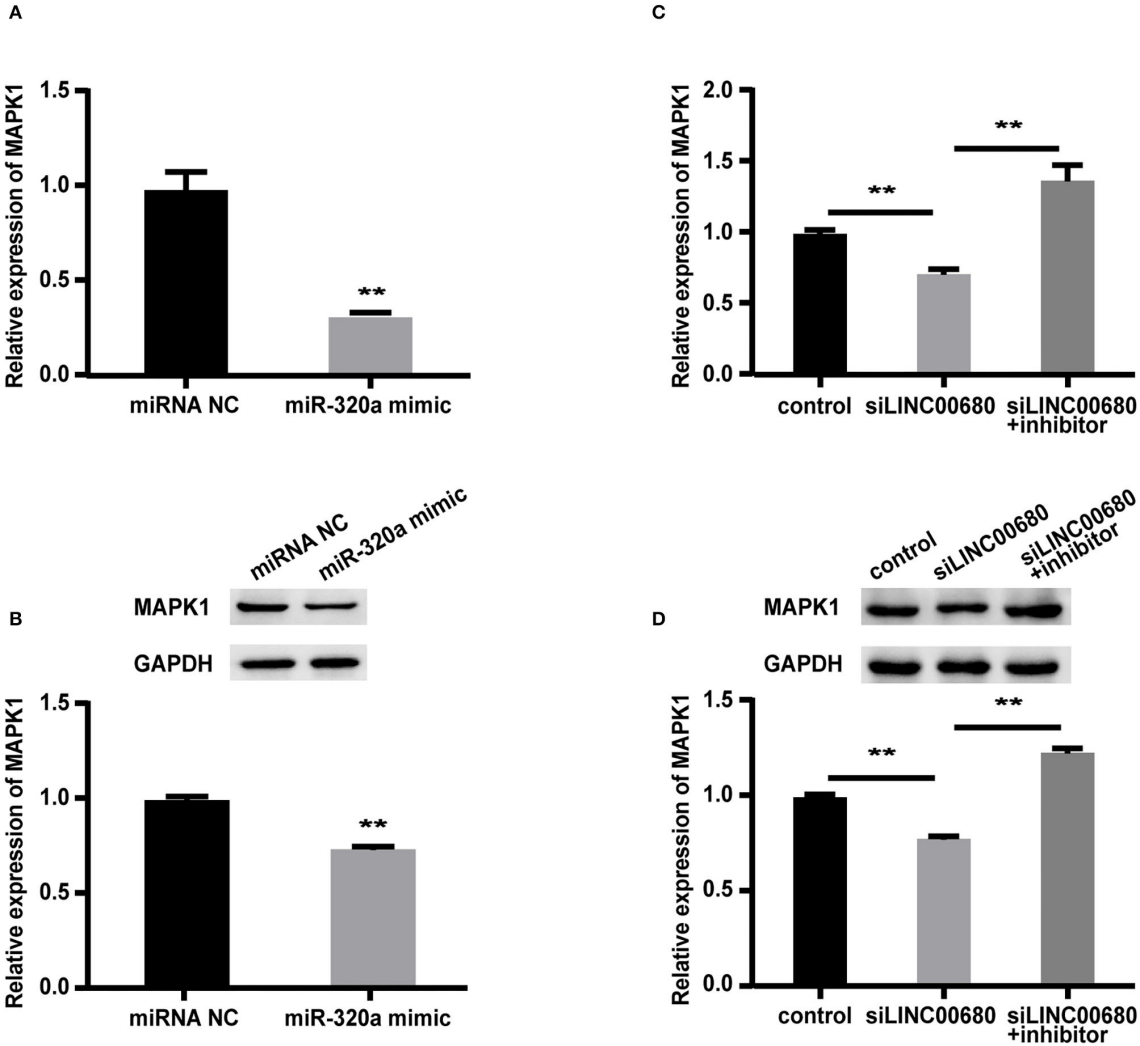
LINC00680 regulated MAPK1 expression by binding miR-320a as a competing endogenous RNA (ceRNA). **(A)** The relative mRNA levels of MAPK1 were determined by real-time PCR after transfection with negative control or miR-320a mimic in Jurkat cells for 48 h **(B)** Relative protein expression levels of MAPK1 were determined by Western blotting after transfection with negative control or miR-320a mimic in Jurkat cells for 48 h **(C)** Relative mRNA levels of MAPK1 were determined by real-time PCR analysis after transfection with negative control, siLINC00680, and siLINC00680 + miR-320a inhibitor in Jurkat cells for 48 h **(D)** Relative protein expression levels of MAPK1 were determined by Western blotting after transfection with negative control, siLINC00680, and siLINC00680 + miR-320a inhibitor in Jurkat cells for 48 h (**p < 0.01).

To detect whether LINC00680 regulates MAPK1 expression by sponging miR-320a, we first transfected Jurkat cells with miR-320a mimics or negative controls to determine the expression levels of MAPK1 protein and mRNA by Western blotting and qRT-PCR, respectively. qRT-PCR was selected to measure the transfection efficiency of the miR-320a mimic (*p* < 0.01; Figure 3A). The analysis indicated that the miR-320a overexpression reduced the MAPK1 expression at the mRNA and protein levels (*p* < 0.01, Figure 4A; *p* < 0.01, Figure 4B), which was consistent with the previous study (1). Then, we transfected Jurkat cells with negative control, siLINC00680, and siLINC00680, along with the miR-320a inhibitor. The MAPK1 mRNA and protein levels were then determined by qRT-PCR and Western blotting. Analysis indicated that the knockout of LINC00680 inhibited the MAPK1 mRNA and protein expression levels in Jurkat cells, while miR-320a inhibitors blocked the siLINC00680-induced reduction in MAPK1 expression (*p* < 0.01, Figure 4C; *p* < 0.01, Figure 4D). These results revealed that LINC00680 regulates the MAPK1 expression by sponging miR-320a in a ceRNA manner.

### LINC00680 Inhibited Apoptosis and Promoted Proliferation by Sponging miR-320a in Jurkat Cells

**Figure 5:**
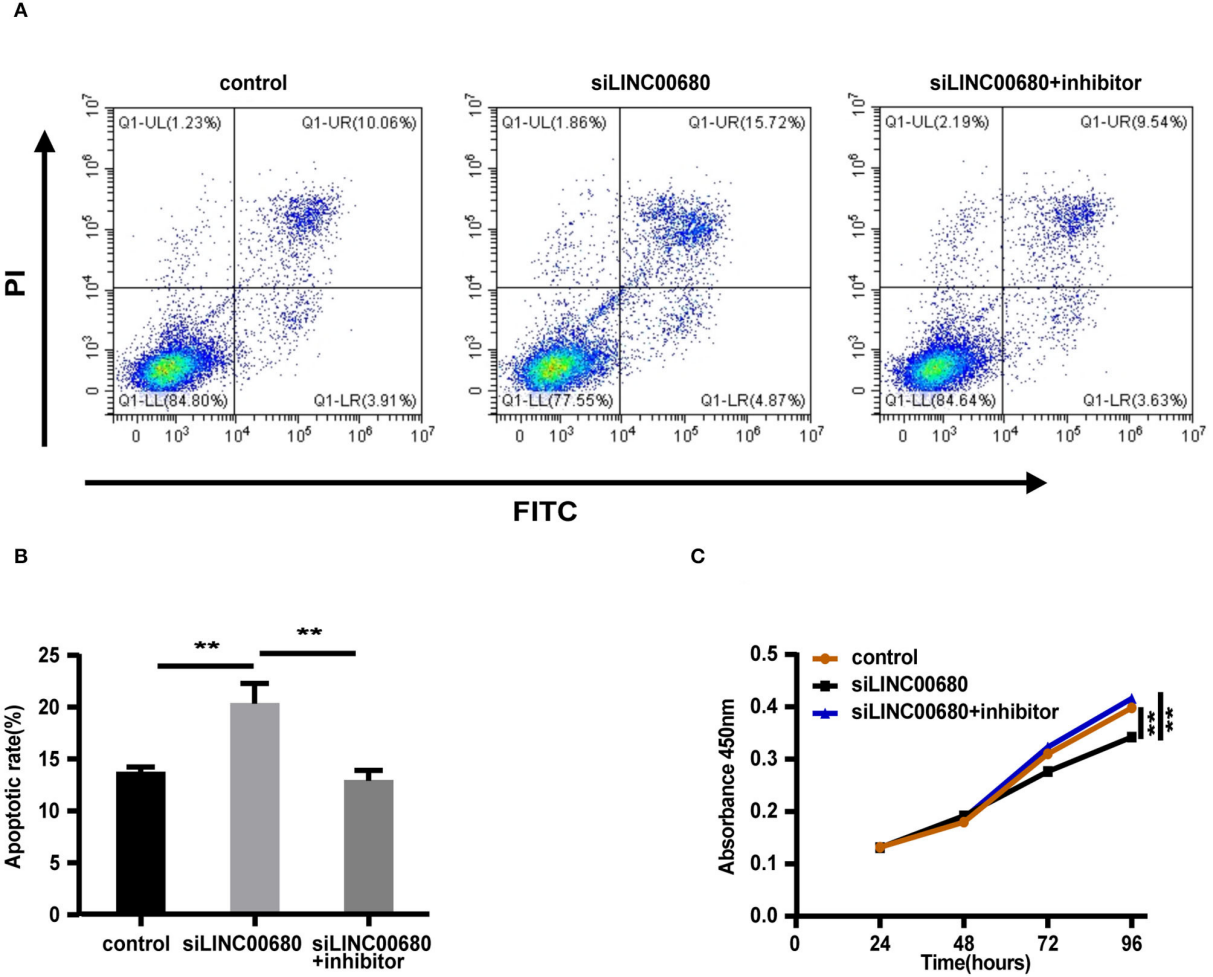
LINC00680 inhibited apoptosis and promoted proliferation by sponging miR-320a. **(A)** After transfecting negative control, siLINC00680, or siLINC00680 + miR-320a inhibitor for 48 h, Jurkat cells were stained with Annexin-V-FITC/propidium iodide (PI) and apoptosis was detected by flow cytometric analysis **(B)** The apoptosis rate of Jurkat cells after transfecting negative control, siLINC00680, or siLINC00680 + miR-320a inhibitor for 48 h **(C)** Cell proliferation was analyzed by Cell Counting Kit-8 (CCK-8) assays after transfecting negative control, siLINC00680, or siLINC00680 + miR-320a inhibitor into Jurkat cells for 24, 48, 72, and 96 h (**p < 0.01).

Since MG is a T-cell-dependent autoimmune disorder, the proliferation and activation of T cells inevitably have a great influence on the occurrence and development of MG. The Jurkat cells were selected for the functional verification of MG (37). To detect whether LINC00680 could affect the proliferation and apoptosis of Jurkat cells by sponging miR-320a, we transfected Jurkat T cells with negative control, siLINC00680, and siLINC00680, along with a miR-320a inhibitor. Cell apoptosis ability was then detected by an Annexin V/PI assay. We found that the proportion of apoptosis increased significantly following the knockdown of LINC00680 in the Jurkat T-cell line. However, the addition of miR-320a inhibitors eliminated this trend (Figures 5A,B). CCK8 assays were selected to measure cell proliferation. Cell proliferation of the siLINC00680 group was lower than that of the control group; cotransfection with the miR-320a inhibitor reversed this effect (Figure 5C). These results revealed that LINC00680 promotes Jurkat T-cell proliferation and inhibits apoptosis by sponging miR-320a. Collectively, our results indicated that LINC00680 could regulate T-cell proliferation and apoptosis by sponging miR-320a and that this process is related to the immunological pathogenesis of MG.

## Discussion

Myasthenia gravis (MG) is an autoimmune disease that seriously threatens the health and even life of patients by causing muscle weakness. However, our ability to diagnose and treat MG remains limited. Therefore, it is vital for us to identify new biomarkers that can determine the severity of MG and serve as effective therapeutic targets. However, biomarkers that meet all the required criteria have yet to be identified. MG has a complex pathogenesis that involves genetic, immune, and environmental factors; collectively, these factors are strongly linked to the susceptibility and development of MG. In recent years, the involvement of lncRNA, as a ceRNA, in the occurrence and development of various diseases has attracted increasing levels of attention. For instance, the lncRNA metastasis associated lung adenocarcinoma transcript 1 (MALAT1) has been shown to act as a ceRNA and regulates the IL-6 expression by sponging miR-1, thus affecting the severity of normal-tension glaucoma (38). These findings improved our understanding of the molecular mechanisms involved and provided a new perspective on how we might identify new biomarkers for MG.

In this study, we systematically detected the potential significance of LINC00680 in MG, which had never been investigated before. Our data displayed that the LINC00680 expression was observably upregulated in MG when compared with controls. When we further investigated the association between the LINC00680 expression levels and the MGC score and the QMG score and between EOMG and LOMG, we found that the LINC00680 expression levels were positively correlated with the MGC and QMG scores; no obvious difference was found between EOMG and LOMG.

Based on these results, we further explored the potential molecular mechanisms underlying the ability of LINC00680 to regulate MG. Bioinformatics analysis predicted that miR-320a may be the miRNA that targets LINC00680 in MG. As a member of the miR-320 family, miR-320a is located on human chromosome 8p21.3 and is closely related to disease progression, tumor invasion, and metastasis (39, 40). The expression of miR-320a was various in different diseases. For example, miR-320a was downregulated in cholangiocarcinoma (41), but it was upregulated in hepatocellular carcinoma (42). It has been reported that miR-320a expression was downregulated in patients with MG (1). Therefore, miR-320a was selected for further analysis. By reviewing the literature, we also found that MAPK1 is a target for miR-320a. Meanwhile, miR-320a is verified to regulate the secretion of MAPK1 in Jurkat cells (1). We also discovered that miR-320a overexpression reduced the MAPK1 mRNA and protein expression levels in Jurkat cells. Then, we detected the miR-320a expression and the MAPK1 expression by qRT-PCR in patients with MG and normal controls. We detected a significant downregulation of miR-320a, along with a significant upregulation of MAPK1, in patients with MG. These discoveries were all consistent with the past publication (1). Moreover, we also found that there was a positive correlation between LINC00680 expression and MAPK1 expression. Therefore, we assumed that LINC00680 regulates the miR-320a/MAPK1 axis as a ceRNA to affect the severity of MG.

To further verify the existence of the LINC0680/miR-320a/MAPK1 axis, we carried out several experiments *in vitro*. First, we transfected miR-320a mimic into Jurkat cells and found that the transfection with miR-320a reduced the expression of LINC00680 in Jurkat cells. Then, luciferase reporter assays were selected to affirm that LINC00680 is a direct target of miR-320a. Finally, we transfected negative control, siLINC00680, and cotransfected siLINC00680 and miR-320a inhibitor into Jurkat cells, respectively. We found that the knockout of LINC00680 inhibited MAPK1 expression at both the protein and mRNA levels; however, these effects could be reversed when Jurkat cells were cotransfected with siLINC00680 and miR-320a inhibitor. In addition, cell proliferation ability and apoptosis ability analysis indicated that the knockdown of LINC00680 could not only inhibit cell proliferation but also promote cell apoptosis. However, these effects could also be reversed by the cotransfection of siLINC00680 and miR-320a inhibitors into Jurkat cells. These results indicated that lncRNA LINC00680 regulates MAPK1 through competitively binding miR-320a as a ceRNA. These findings increase our knowledge of the specific mechanisms underlying MG.

Mitogen-activated protein kinase 1 (MAPK1) is a vital member of the MAPK family. The activation of MAPK is closely related to the transcription and translation of cytokines (43). Furthermore, MAPK signaling pathways are essential for the synthesis and amplification of inflammatory factors (44). These inflammatory factors have been indicated to have a significant function in the immunological pathogenesis of MG (45). A study has shown that IL-2 could affect the pathogenesis of MG (46). It has also been reported that IFN-γ could affect the severity of experimental MG (14). A previous study has shown that miR-320a can repress the production of IFN-γ and IL-2 by directly inhibiting MAPK1 expression in MG (1). Therefore, these findings confirm that LINC00680 may affect the severity of MG by regulating the miR-320a/MAPK1 axis.

This is the first study to investigate the association between the LINC00680 expression and the severity of MG and, therefore, demonstrate the biological function and molecular mechanisms of LINC00680 in MG, which regulates the expression of MAPK1 by sponging miR-320a. However, several limitations to this study need to be addressed. First, the sample size of this study is not large enough and a larger sample size is needed to further confirm the results of this study. Second, more recovery experiments should be added to further confirm that LINC00680 sponges miR-320a affected T-cell proliferation and apoptosis by regulating MAPK1.

## Conclusion

Our data demonstrated that LINC00680 may be associated with the severity of MG and act as a ceRNA. In this study, we identified LINC00680 as a new biomarker for the diagnosis, development, and treatment of MG.

## Data Availability Statement

The raw data supporting the conclusions of this article will be made available by the authors, without undue reservation.

## Ethics Statement

The studies involving human participants were reviewed and approved by the Ethics Committee of The Second Affiliated Hospital of Harbin Medical University. The patients/participants provided their written informed consent to participate in this study.

## Author Contributions

LW and JW conceived and designed the study. LLiu performed the experiments, analyzed the data, and drafted the manuscript. HZ, XL, LLi, and TW provided intellectual support and contributed reagents and analytical tools for the study. LLi, QL, TW, ZC, and HG provided guidance on data analysis. SL, XW, SX, and TY revised the manuscript. All authors have read and approved the submitted version of the manuscript.

## Funding

This study was supported by the National Natural Science Foundation of China (Grant No: 81820108014, 82171396, 82071407, and 81901277), the National Key Research and Development Project (Grant No: 2018YFE0114400), and the Heilongjiang Provincial Natural Science Foundation (Grant No: LH2019H086 and YQ2021H012).

## Conflict of Interest

The authors declare that the research was conducted in the absence of any commercial or financial relationships that could be construed as a potential conflict of interest.

## Publisher's Note

All claims expressed in this article are solely those of the authors and do not necessarily represent those of their affiliated organizations, or those of the publisher, the editors and the reviewers. Any product that may be evaluated in this article, or claim that may be made by its manufacturer, is not guaranteed or endorsed by the publisher.
