# Supplementary material for: LncRNA LINC00680 Acts as a Competing Endogenous RNA and Is Associated With the Severity of Myasthennia Gravis
Source: Front Neurol. 2022 Jun 21;13:833062. doi: 10.3389/fneur.2022.833062 (PMC9253289; doi:10.3389/fneur.2022.833062)
Supplement: Supplementary file 1 [file Table_1.pdf]

Supplementary Table 1. Detailed information on the severity of MG patients

NO.	Quantitative Myasthenia Gravis Score (QMGs)	Myasthenia Gravis Composite (MGC)
1	13	25
2	17	10
3	12	12
4	15	9
5	6	7
6	7	11
7	11	13
8	6	10
9	14	20
10	8	9
11	5	5
12	6	9
13	13	19
14	10	14
15	6	7
16	8	12
17	4	9
18	7	7
19	6	7
20	8	8
21	2	6
22	9	12
23	6	7
24	12	16
25	6	8
26	3	3
27	2	4
28	8	12
29	5	7
30	7	7
31	7	9
